# Risk for intravesical recurrence of bladder cancer stratified by the results on two consecutive UroVysion fluorescence in situ hybridization tests: a prospective follow-up study in Japan

**DOI:** 10.1007/s10147-020-01634-9

**Published:** 2020-03-03

**Authors:** Atsushi Ikeda, Takahiro Kojima, Koji Kawai, Shiro Hinotsu, Naoto Keino, Kenichiro Shiga, Hideaki Miyake, Yasuyoshi Miyata, Yutaka Enomoto, Fumitaka Shimizu, Satoshi Anai, Hideyasu Matsuyama, Chieko Suzuki, Yusuke Kanimoto, Keisuke Shigeta, Seiji Naito, Hideyuki Akaza, Hiroyuki Nishiyama

**Affiliations:** 1 Department of Urology, University of Tsukuba, 1-1-1 Tennodai, Tsukuba, Ibaraki 3058575 Japan; 2 Department of Biostatistics, Sapporo Medical University, Sapporo, Japan; 3 Tsukuba Clinical Research and Development Organization (T-CReDO), University of Tsukuba, Tsukuba, Japan; 4 Department of Urology, Harasanshin Hospital, Fukuoka, Japan; 5 Department of Urology, Hamamatsu University School of Medicine, Hamamatsu, Japan; 6 Department of Urology, Nagasaki University, Nagasaki, Japan; 7 Department of Urology, Mitsui Memorial Hospital, Tokyo, Japan; 8 Department of Urology, Juntendo University, Tokyo, Japan; 9 Department of Urology, Nara Medical University, Kashihara, Japan; 10 Department of Urology, Yamaguchi University, Ube, Japan; 11 Department of Urology, Anjo Kosei Hospital, Anjo, Japan; 12 Department of Urology, Chutoen General Medical Center, Kakegawa, Japan; 13 Department of Urology, Keio University, Tokyo, Japan; 14 Strategic Investigation On Comprehensive Cancer Network, The University of Tokyo, Tokyo, Japan

**Keywords:** UroVysion, Non-muscle-invasive bladder cancer, Intravesical recurrence

## Abstract

**Background:**

A previous comparative study in Japan has demonstrated that the two consecutive UroVysion tests are useful tools to detect the presence of bladder cancer during follow-up after transurethral resection, but they also presented their high rates of false-positive results. Here, we aimed to evaluate the relationship between the UroVysion tests and subsequent intravesical recurrence.

**Methods:**

In the previous study, patients without bladder cancer during the first analysis showed the same examination set repeated 3 months later as the second analysis. In this follow-up study, 326 patients showed negative findings confirmed on cystoscopy during the second UroVysion test. Recurrence-free survival was assessed using a median follow-up of 27 months.

**Results:**

In the two consecutive UroVysion tests, 214 patients (65.6%) showed negative UroVysion results in both tests, whereas 91 presented a positive result on either tests and 21 patients presented positive results in both tests. During the follow-up, 40 patients (12.3%) had an intravesical recurrence with non-muscle-invasive bladder cancer. The recurrence rates in patients with negative results in both tests, those with one positive result in either tests, and those with positive results in both tests were 8.4%, 16.5%, and 33.3%, respectively. The multivariate analysis indicated that the history of bladder cancer and the consecutive UroVysion test pattern were independent risk factors for recurrence.

**Conclusions:**

Our data confirmed the effectiveness of two consecutive UroVysion tests in predicting intravesical recurrence after TURBT. Further prospective studies would help determine an appropriate interval for cystoscopy follow-up.

## Introduction

In 2018, bladder cancer (BC) was the sixth most common cancer in men and the 17th most common cancer in women worldwide, and 550,000 new cases occur each year [1]. Non-muscle-invasive bladder cancer (NMIBC) accounts for 75% of BC cases. In NMIBC, 50–80% of patients suffered from intravesical recurrence; of these, 15–25% will develop muscle-invasive BC. The high rate of intravesical recurrence and progression leads to the deterioration of the quality of life of patients [2].

Cystoscopy and urinary cytology are the most widely used examinations for monitoring BC patients. However, the sensitivity of urinary cytology, especially that for the low-grade tumor, is not sufficient. In this point, recently, the UroVysion test (UroVysion Bladder Cancer Kit:Abbot Molecular, Des Plaines IL), a multicolor fluorescence in situ hybridization technique that detects aneuploidy of chromosomes 3, 7, and 17 and the loss of 9p21 locus, showed promising results as a more sensitive tool for the initial diagnosis and monitoring in several studies conducted in Western countries [3, 4]. Moreover, in Japan, a large comparative study of two consecutive UroVysion tests enrolled 486 BC patients and demonstrated higher sensitivity of the tests than did urine cytology [5]. The UroVysion test had 50.0% (95% CI 35.2–64.8%) sensitivity and 72.4% (68.3–76.8%) specificity. Urine cytology had 4.5% (0.0–10.7%) sensitivity and 99.8% (99.3–100.0%) specificity. However, in this study, BC was not detected in 116 patients with positive UroVysion test results (false-positive rate 27.6%). This may be due to the low specificity of the UroVysion test. On the other hand, there is a possibility that the positive UroVysion test reflects the presence of invisible precursor lesions in some patients. Thus, we aimed to evaluate the relationship between the previous UroVysion tests and subsequent intravesical recurrence.

## Patients and methods

### Study design

**Fig. 1:**
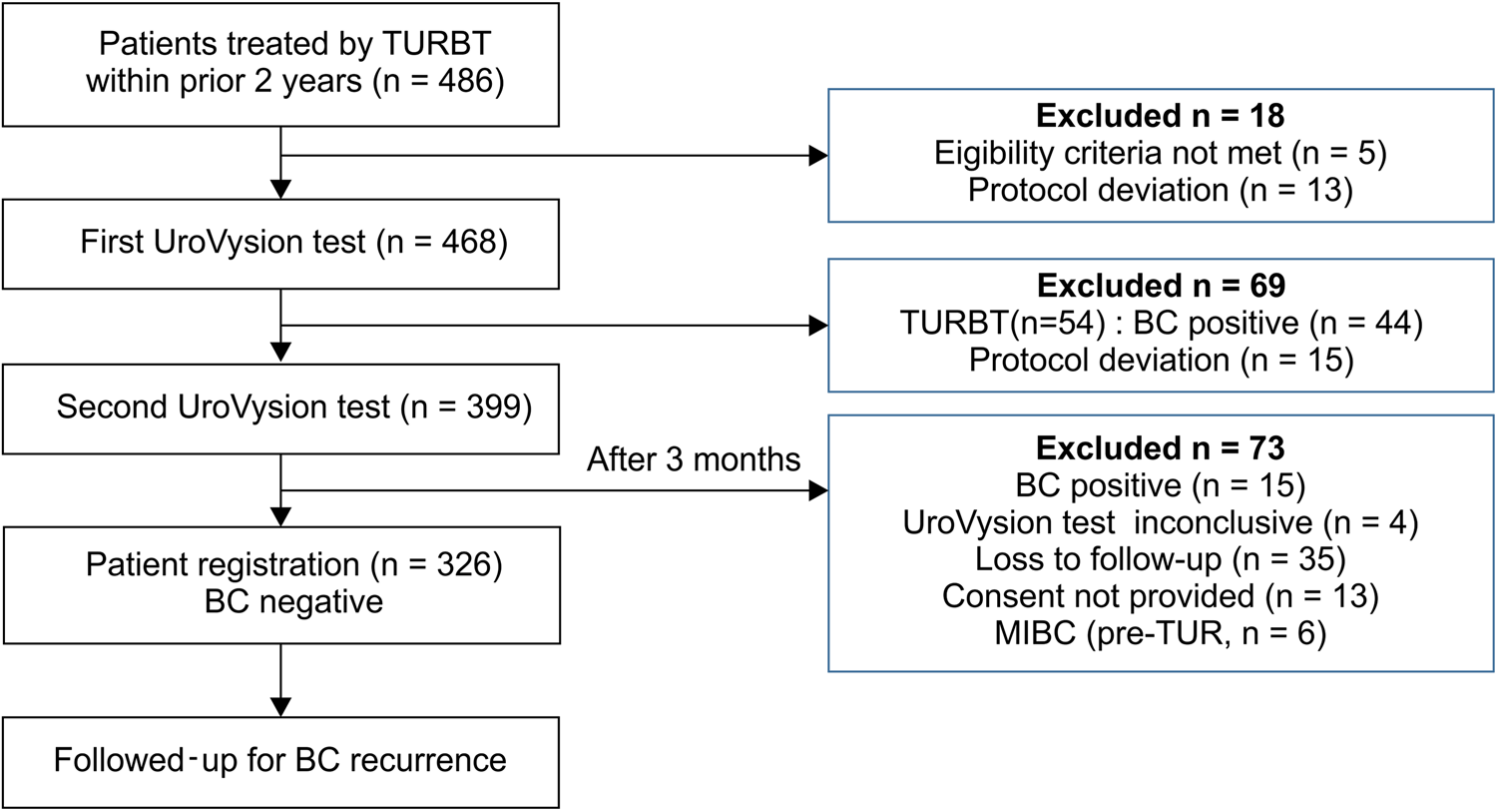
Flow diagram of patient enrollment. *MIBC*muscle-invasive bladder cancer,*TURBT*transurethral resection, *BC* bladder cancer

A total of 486 BC patients treated with transurethral resection (TURBT) within the prior 2 years were enrolled in the previous comparative study of two consecutive UroVysion tests. Among them, two consecutive results were obtained in 399 patients. In patients who showed no suspicious findings of recurrent BC during the first analysis, a second set of the UroVysion test and urine cytology was performed 3 months after the first set. Patients who showed suspicious findings in the second analysis underwent histological examination of the biopsy or transurethral resection, as in the first analysis. BC was detected in 15 patients at the second UroVysion test, and additional 6 patients developed MIBC before initiation of the present study. As shown in Fig. 1, 52 patients were excluded from the present study, because they were lost to follow-up (35 patients), had a disagreement with the study (13 patients), and had inconclusive UroVysion test (4 patients). Resultantly, 326 patients with negative cystoscopy and cytology results were enrolled in the present study. Patients were followed for BC recurrence according to the protocol of each participating institution. The follow-up period was defined as the time from the second UroVysion test until the date of the last cystoscopy or the date when BC was diagnosed by cystoscopy or cytology. The median follow-up period was 27 months (range 1–36.4 months). All BC recurrences were pathologically confirmed. This study was conducted in accordance with the ethical principles of the 2013 Declaration of Helsinki and was approved by the Institutional Review Board of the University of Tsukuba Hospital, which is the representative medical organization (Approval number: H28-184). We obtained written informed consent from patients and provided the option of declining the invitation to participate in the study.

### Statistical analysis

Patient characteristics were compared using the Pearson's chi-square test. Recurrence-free survival (RFS) curves were estimated by the Kaplan–Meier method and compared using a log-rank test. The level of significance was set at *P* < 0.05. The relative influence of different clinical factors on RFS was estimated using Cox’s proportional hazards model with multiple variables. We enlisted a purposeful selection of variables in the analysis, used a *P* value of < 0.05, and excluded variables that were not statistically significant. Statistical analysis was performed using JMP 10.0.2 software (SAS Institute, Cary, NC).

## Results

The characteristics of 326 patients are summarized in Table 1. The median age was 71 years (range 26–91 years). The history of BC was primary in 226 patients (69.3%) and recurrent in 100 patients (30.7%). The initial T stage before study enrollment was Ta in 58.9% of patients, T1 in 31.9%, and Tis in 7.1%. The carcinoma in situ (CIS) was confirmed in 71 patients (21.8%), including those with concurrent CIS. In the two consecutive UroVysion tests, 214 patients (65.6%) presented negative results for both tests, whereas 91 patients (27.9%) presented positive results in one of the tests, and 21 patients (6.4%) showed positive results in both tests. As shown in Table 1, there was no significant difference in the distribution of clinical characteristics among patients with negative results in both tests, those with one positive result in one of the tests, and those with positive results in both tests.

**Table 1:** Patient characteristics

	*N*	%	Negative results in both tests (*n* = 214)		Positive result in one of the tests (*n* = 91)		Positive results in both tests (*n* = 21)	
			*n*	%	*n*	%	*n*	%
Male	281	86.2	184	86.0	80	87.9	17	81.0
Female	45	13.8	30	14.0	11	12.1	4	19.0
Age, years								
Median (range)	71 (26–91)		70 (26–90)		72 (40–91)		69 (40–85)	
History of bladder cancer								
Primary	226	69.3	151	70.6	61	67.8	14	66.7
Recurrent	100	30.7	63	29.4	30	32.2	7	33.3
No. of lesions								
Single	151	46.3	97	45.3	44	48.3	10	47.6
Multiple	136	41.7	90	42.1	41	45.1	5	23.8
Cannot be evaluated	39	12.0	27	12.6	6	6.6	6	28.6
Grade^a^								
Low grade	138	41.9	97	45.3	30	33.0	11	52.4
High grade	141	44.0	90	42.1	44	48.3	7	33.3
Cannot be evaluated	47	14.1	27	12.6	17	18.7	3	14.3
TNM stage								
Ta	192	58.9	129	60.3	48	52.7	15	71.4
T1	104	31.9	68	31.8	33	36.3	3	14.3
Tis	23	7.1	14	6.5	6	6.6	3	14.3
Cannot be evaluated	7	2.1	3	1.4	4	4.4	0	0
Presence of CIS								
No	255	78.2	168	78.5	70	76.9	17	81.0
Yes	71	21.8	46	21.5	21	23.1	4	19.0
Smoking								
Current	60	18.4	39	18.2	17	18.7	4	19.1
Former	112	34.4	71	33.2	31	34.0	10	47.6
Never	91	27.9	62	29.0	24	26.4	5	23.8
Unknown	63	29.3	42	19.6	19	20.9	2	9.5

There was no significant difference in the distribution of clinical characteristics

*CIS* carcinoma in situ, *TNM* tumor-node-metastasis

^a^1973 World Health Organization classification

**Fig. 2:**
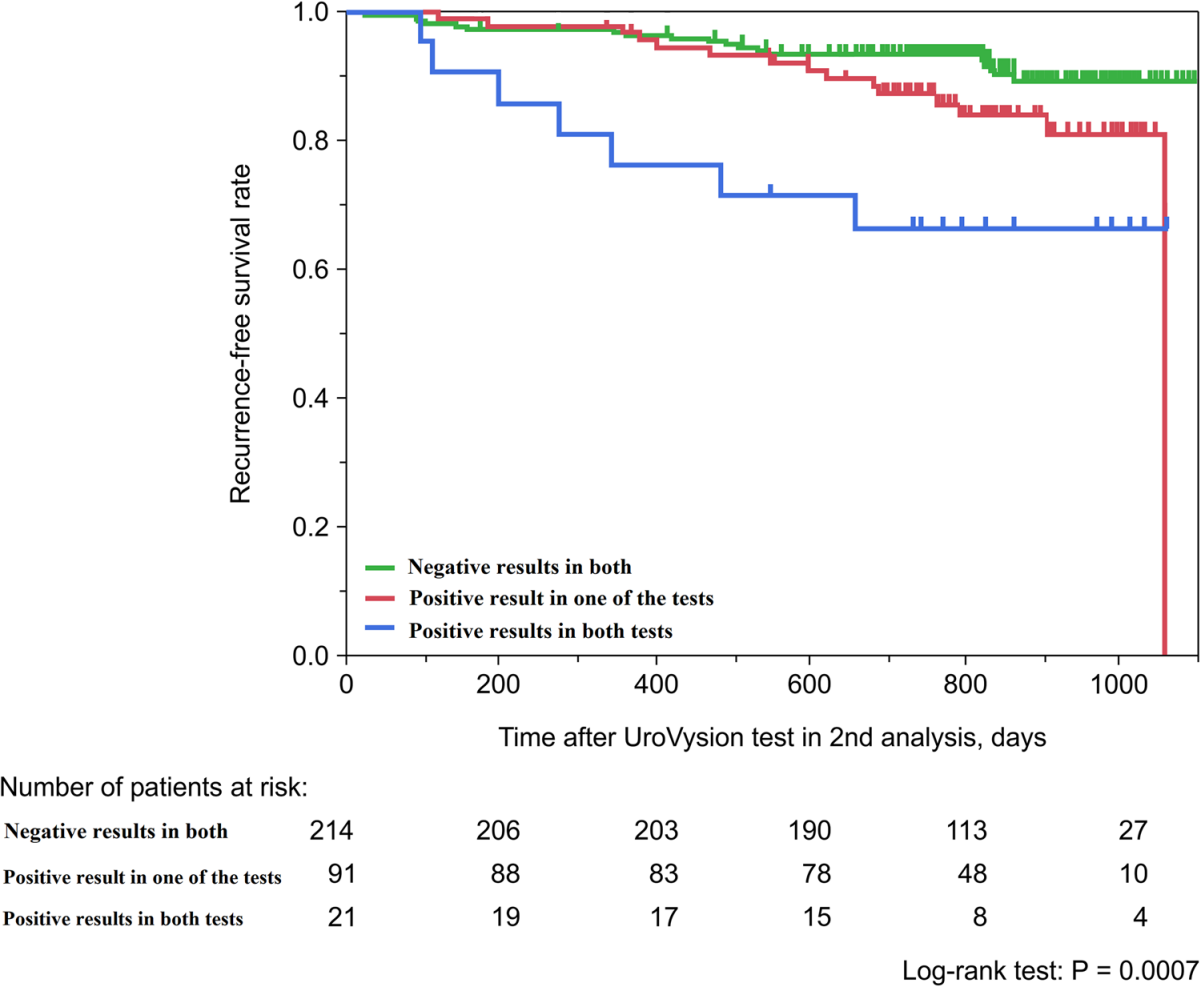
Recurrence-free survival rate stratified by the pattern of the results of the two consecutive UroVysion tests

Intravesical BC recurrence was observed in 40 (12.3%) out of the 326 patients during the follow-up period (Table 2). The pathological diagnoses of recurrent tumors were low-grade Ta tumor in 20 patients (6.1%), high-grade Ta tumor, T1 tumor, or CIS in 15 patients (4.6%), and ≥ T2 tumor in three patients (0.9%). Eighteen out of 214 patients (8.4%) with negative results for both UroVysion tests had tumor recurrence. The recurrence rates of patients with positive results in one of the tests and positive results in both tests were higher (16.5% and 33.3%, respectively). Resultantly, in total, the 2-year RFS rate of the patients with positive results in both tests was 71.4%, which was significantly lower than that of patients with negative results in both tests (93.5%) and those with positive results in one of the tests (87.9%) (Fig. 2, *P* = 0.0007). The median interval to recurrence in patients with positive results in both tests was 9.0 months, which was shorter than that of patients with positive results in one of the tests (19.6 months) and those with negative results in both tests (23.5 months).

**Table 2:** Recurrence rate and pathological diagnosis of recurrent tumor according to the UroVysion test pattern

	Total	Negative results in both tests	Positive result in one of the tests	Positive results in both tests
No. of patients	326	214	91	21
No. of recurrences (%)	40 (12.3)	18 (8.4)	15 (16.5)	7 (33.3)
TNM stage, Grade (%)				
Ta low	20 (50)	9 (50)	8 (53.3)	3 (42.9)
Ta high/T1/Tis	15 (37.5)	8 (44.4)	4 (26.7)	3 (42.9)
T2 or greater	3 (7.5)	1 (5.6)	1 (0.7)	1 (14.2)
Cannot be evaluated	2 (5)	0	2 (1.3)	0

In the Cox proportional hazards model, as shown in Table 3, the univariate analysis showed that the history of BC, T stage, and consecutive UroVysion test pattern were associated with a higher risk for subsequent recurrence. The other variables were not significant predictors. On the basis of the results of the univariate analysis, we performed a multivariate analysis using the following factors: the history of BC, T stage, and consecutive UroVysion test pattern. The results indicated that the history of BC and consecutive UroVysion test patterns were independent risk factors for recurrence.

**Table 3:** Univariate and multivariate Cox proportional hazards regression analyses for recurrence after the UroVysion test in the second analysis

Variable (*n*)	Number of patients	Univariate		Multivariate	
		HR (95% CI)	*P* value^*^	HR (95% CI)	*P* value^*^
Age, years					
< 70	160	1 (reference)	0.5422		
71 <	166	0.824(0.437–1.53)			
Sex					
Female	45	1 (reference)	0.166		
Male	281	2.10 (0.758–8.71)			
History of bladder cancer					
Primary	226	1 (reference)	0.00250	1 (reference)	0.0069
Recurrent	100	2.63 (1.41–4.94)		2.39 (1.27–4.51)	
No. of lesions					
Single	151	1 (reference)	0.0650		
Multiple	136	1.87 (0.926–3.94)			
T stage					
Ta	192	1 (reference)		1 (reference)	
T1	104	0.502 (0.200–1.11)	0.0899	0.566 (0.224–1.26)	0.1708
Tis	23	2.57 (1.02–5.70)	0.0459	2.16 (0.848–4.82)	0.1012
Grade					
Low	138	1 (reference)	0.214		
High	141	0.809 (0.389–1.67)			
Presence of CIS					
No	255	1 (reference)	0.346		
Yes	71	1.41 (0.674–2.74)			
Postoperative intravesical therapy					
No	97	1 (reference)	0.866		
BCG	116	0.831 (0.376–1.85)			
Chemotherapy	113	0.990 (0.463–2.16)			
Smoking					
Never	91	1 (reference)	0.640		
Former	112	1.34 (0.608–3.08)			
Current	60	1.39 (0.554–3.46)			
Combination of two consecutive analyses of UroVysion tests					
Negative results in both tests	214	1 (reference)		1 (reference)	
Positive result in one of the tests	91	2.02 (1.00–4.02)	0.0488	2.06 (1.01–4.13)	0.0467
Positive results in both tests	21	4.71 (1.83–10.8)	0.0024	4.15 (1.59–9.65)	0.0051

*P < 0.05 was considered statistically significant

## Discussion

In Japan, a prospective comparative study showed that the UroVysion test provided higher sensitivity than urine cytology in detecting BC during follow-up after TURBT [5]. Based on these outcomes, UroVysion was approved in Japan in 2017 as a genetic test for the diagnosis of BC recurrence. Still, the indication is limited to patients having CIS and also the test frequency of two times within 2 years after TURBT. Despite the high sensitivity, a Japanese study also presented the high false-positive rate of the UroVysion test. Generally, false-positive results in the UroVysion test can be due to the umbrella cells, often appearing as chromosome tetraploid [6], or heteroploidy appearing due to human polyomavirus infection [7]. Although several kinds of mechanism for true false-positive results have existed, there is a possibility that the positive UroVysion test results reflect the presence of invisible precursor lesions in some patients.

To test the hypothesis, there are several long-term follow-up studies after the UroVysion test. These studies showed that patients with positive UroVysion test have a significantly higher recurrence rate. However, previous studies were limited, because they had a relatively small cohort size, and more importantly, the UroVysion results were disclosed to the attending physician [4, 8, 9]. In this point of view, the present study involved 326 patients, and the results of the UroVysion tests were not revealed to the attending physician. Furthermore, the UroVysion test was conducted twice with an interval of approximately 3 months, which confirmed that the recurrence rate increased depending on the test result pattern, with the rate increasing in order from patients with negative results in both tests (8.4%), those with positive results in one of the tests (16.5%), and those with positive results in both tests (33.3%). Resultantly, the 2-year RFS rate of those with positive results in both tests was 71.4%, which was significantly lower than that of patients with negative results in both tests (93.5%) and those with positive results in one of the tests (87.9%). The multivariate analysis indicated that T stage and the consecutive UroVysion test pattern were independent risk factors for recurrence. Given the positive rates of urinary cytology were relatively low in terms of recurrence without CIS, the two consecutive UroVysion tests are considered to be useful tools for predicting this type of recurrence.

A cystoscopy is an essential tool for BC follow-up, but there has always been room for debate on the appropriate frequency of cystoscopic examinations. The present study showed that the recurrence rate was higher in patients with positive results in both tests, and these patients developed recurrence with a shorter interval (Fig. 2). The finding suggests the possibility of precision management with the setting of the intervals of cystoscopy based on the results of the UroVysion test.

This study has several limitations. First, patients were followed for BC recurrence according to the protocol of each participating institution. Second, almost all patients underwent conventional white light cystoscopy, but not a photodynamic diagnosis or narrow-band imaging examination during follow-up. Therefore, further study is needed to clarify the role of UroVysion tests in the era of high-resolution endoscopes. Despite these limitations, our data confirmed the effectiveness of two consecutive UroVysion tests in predicting intravesical recurrence after TURBT. Further prospective studies would help determine an appropriate interval for cystoscopy follow-up.
